# Study on Interfacial Bonding Properties of NiTi/CuSn10 Dissimilar Materials by Selective Laser Melting

**DOI:** 10.3390/mi13040494

**Published:** 2022-03-22

**Authors:** Changhui Song, Zehua Hu, Yunmian Xiao, Yang Li, Yongqiang Yang

**Affiliations:** School of Mechanical and Automotive Engineering, South China University of Technology, Guangzhou 510640, China; 201920100681@mail.scut.edu.cn (Z.H.); 201910100307@mail.scut.edu.cn (Y.X.); meyangli@scut.edu.cn (Y.L.); meyqyang@scut.edu.cn (Y.Y.)

**Keywords:** NiTi alloy, CuSn10 alloy, selective laser melting, dissimilar materials, interfacial bonding properties

## Abstract

The dissimilar materials bonding of NiTi alloy with shape memory effect (SME) and CuSn10 alloy with good ductility, electrical conductivity, and thermal conductivity can be used in aerospace, circuits, etc. In order to integrate NiTi and CuSn10 with greatly different physical and chemical properties by selective laser melting (SLM), the effects of forming interlayers with different SLM process parameters were explored in this study. The defects, microstructure, and component diffusion at the interface were also analyzed. Columnar grain was found along the molten pool boundary of the interfacial region, and grains in the interfacial region were refined. Elements in the interfacial region had a good diffusion. Phase identifying of the interface showed that Ni_4_Ti_3_ was generated. The analysis showed that the columnar grain, refined grains in the interfacial region, and a certain amount of Ni_4_Ti_3_ could strengthen the interfacial bonding. This study provides a theoretical basis for forming NiTi/CuSn10 dissimilar materials structural members.

## 1. Introduction

SLM is the most common metal additive manufacturing technology. In this technology, a high energy density laser is utilized as a heat source. The laser spot is concentrated in a very small range to selectively melt the spherical metal powder on the powder bed, by which parts with quite complex shapes can be obtained [1]. It is widely used in aerospace and medical fields because of high forming accuracy [2,3,4]. However, SLM is mainly used to manufacture single metal parts.

SLM of single material has been unable to meet the performance requirements of people with the continuous development of its commercial application. Therefore, the research of SLM of two or even multiple materials has appeared in recent years. Chen et al. [5] studied the forming of CuSn10/316 L dissimilar materials and found that the generation of interfacial defects was relevant to process parameters of the interface. The defects near the interface were the main factors affecting the bonding strength. Finally, dissimilar materials’ tensile parts with tensile strength close to that of CuSn10 single alloy tensile part were formed successfully. Liu et al. [6] formed 18Ni300 maraging steel on traditional machined Cr8Mo2SiV cold work die steel by SLM. They found that a toothed mosaic structure could be formed at the interfacial area, which could promote interfacial bonding. Tan et al. [7] studied the manufacturing of maraging steel/copper dissimilar materials by the combination of selective laser melting and subtractive process. They found that the improvement of interfacial bonding performance was related to the intense Marangoni flows at the interface. This kind of manufacturing can combine the advantages of multiple metal materials, and therefore is very promising.

Shape memory alloy (SMA) refers to the alloy with SME due to the reversibility of phase transition [8], among which NiTi alloy is the most widely used. It has been applied in sensors, actuators, medical devices, and many other fields [9,10]. The integrated forming of NiTi alloy and other materials can combine the functionality of NiTi with the excellent properties of other materials, thus having broad application prospects. Some studies on the combination of NiTi alloy with other alloys have been conducted in recent years. Li et al. [11] studied the laser welding process of NiTi SMA wire and stainless-steel wire using nickel as the interlayer. It found that appropriate Ni interlayer thickness could reduce the brittle intermetallic compounds and pores, thus improving the bonding properties. Gao et al. [12] proposed a laser welding method using Nb/Cu multi-interlayer. Through a single pass welding, a hybrid joint composed of three metallurgical bonding zones was obtained according to different welding mechanisms. This completely prevented the formation of Ti-Fe and Ti_x_Ni_y_ intermetallic compounds, and thus significantly improved the mechanical properties of the joint. Panton et al. [13] studied the laser welding of Nitinol wire and MP35N wire and found that the position of the laser beam controlled the composition of the molten pool, while the intensity of the laser beam affected the size and mixing of the molten pool. These variables greatly influenced the hardness and cracking sensitivity of the interfacial region, and thus seriously affected the bonding strength. At present, one of the research hotspots of NiTi alloy-related dissimilar materials is the combination of Ti6Al4V and NiTi alloy. Because it can be used in biomedical and aerospace fields, thus it is quite promising. The main problem of NiTi bonding with Ti6Al4V is that brittle intermetallic compounds can be easily formed during the process, which affects the bonding properties. Oliveira et al. [14] used niobium as an interlayer to prevent the generation of brittle phase during laser welding of NiTi and Ti6Al4V. The melting temperature of the niobium interlayer is much higher than that of the matrix material. As a result the bulk niobium did not melt during the joining process, which played a role in the diffusion barrier between these two materials. It ensured that no cracks in the welds and brittle intermetallic compounds were discovered. Zoeram et al. [15] utilized thin copper as an interlayer to conduct laser welding of NiTi and Ti6Al4V. It found that selecting an appropriate interlayer thickness could inhibit the formation of Ti-Cu intermetallic compounds and shrinkage cavities, thus improving the interfacial bonding properties. Xie et al. [16] studied the ultrasonic spot welding of Ti6Al4V/NiTi dissimilar joints with Al coating as an interlayer and found that the firm bonding of Ti6Al4V/NiTi dissimilar materials can be realized by selecting appropriate welding pressure. Bartolomeu et al. [17] fabricated NiTi-Ti6Al4V multi-material cellular structures with a Ti6Al4V inner region and a NiTi outer region by SLM. The bonding between NiTi and Ti6Al4V in the transition region was declared to be successful by morphology analysis. There were also some studies on other Ni-based alloys. Tabaie et al. [18,19,20] studied joining SLM Inconel 718 with forged AD730 TM Nickel-based superalloy using linear friction welding (LFW). Joints free of micro-porosity, micro-cracks, and oxides were obtained. It was found that the strength of AD730 TM alloy depended significantly on the grain size while that of SLM Inconel 718 was dominated by shape (or size) and the presence of secondary phases (γ′/ γ″ and Laves). However, both the size and the volume fraction of all phases affected the hardness and mechanical properties of the joint. After welding, a new post-weld heat treatment cycle was designed, resulting in uniform hardness across the dissimilar joint. It can be found from the above research that welding is a quite commonly used technology for the combination of NiTi alloy and other Ni-based alloys related dissimilar materials, in which an interlayer is often applied. SLM technology is seldom used for the combination. This is partly due to the forming of the NiTi alloy by SLM is still immature [21]. Conversely, the studies about the combination of two kinds of materials using SLM are still in their nascence; there are still many mechanisms that need to be researched.

CuSn10 is a type of tin bronze with good ductility, electrical conductivity, and thermal conductivity. NiTi/CuSn10 dissimilar materials parts can combine the excellent shape memory performance of NiTi alloy with the advantages of CuSn10, which has a wide application prospect. The technical experts of NASA are developing a sort of smart radiator that controls heat-loss rate flexibly by deforming. The thermosensitive materials can be utilized to realize the change of shape. When the thermosensitive material experiences the temperature change, the radiator can automatically change its shape to dissipate heat or store heat. This expectation can be achieved by using NiTi alloy to achieve the shape change and using CuSn10 alloy to dissipate heat. As for electrical conductivity, an intelligent fuse can be realized based on the mobile deformation of NiTi alloy and the high electrical conductivity of CuSn10 alloy, which can control the circuit on and off in real-time, according to the temperature to achieve protection. There are also many other possibilities. However, there are few studies on the bonding between NiTi alloy and CuSn10 alloy. This is because the interface is a part of the dissimilar materials parts at which defects are most likely to occur and a significant factor determining the reliability of the whole part. The bonding properties of the interface are mainly affected by the differences between the physical and chemical properties of the two materials, which were found to be large [6,22,23]. In this study, the effects of adding CuSn10 interlayers with different process parameters between NiTi alloy matrix and CuSn10 alloy by SLM on the defects, microstructure, phases, and properties at the interface were investigated, providing a theoretical basis for forming NiTi/CuSn10 dissimilar materials structural parts. The results showed that appropriate Ni_4_Ti_3_ content and columnar grain at the interface can be realized by selecting appropriate parameters, the combination of which can improve the interfacial bonding properties. Refined grains in the interfacial region can also play a strengthening role.

## 2. Materials and Methods

### 2.1. Experimental Equipment and Materials

**Figure 1:**
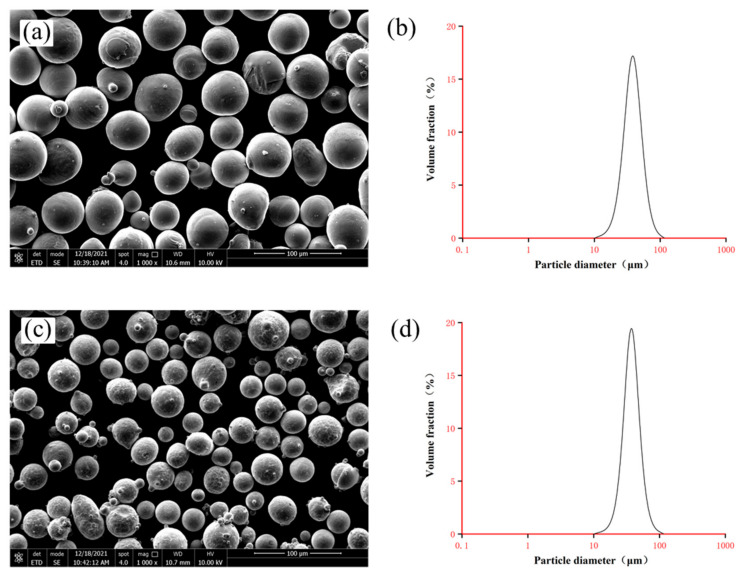
(**a**) SEM image of equiatomic NiTi50 powder (1000×); (**b**) particle size distribution for equiatomic NiTi50 powder; (**c**) SEM image of CuSn10 powder (1000×); (**d**) particle size distribution for CuSn10 powder.

The SLM equipment used in this study is the Dimetal-100H device jointly developed by the additive manufacturing team of South China University of Technology and Laseradd Technology (Guangzhou, China) Co Ltd. The main equipment parameters were shown in Table 1. The materials used in this study were equiatomic NiTi50 powders and CuSn10 powders. Equiatomic NiTi50 powders were manufactured by Electrode Induction Melting Gas Atomization (EIGA) technology (Beijing Avimetal Powder Metallurgy Technology Co., Ltd., Beijing, China) and the particle size ranges from 15 to 53 μm (degree of sphericity ≈ 90%). CuSn10 powders were also manufactured by EIGA technology (Dongguan Hyper Tech Co Ltd., Dongguan, China) and the particle size ranges from 13 to 53 μm (degree of sphericity > 90%). Inductive Coupled Plasma Emission Spectrometer (ICP) was used to measure the chemical compositions which were listed in Table 2 and Table 3. The scanning electron microscope (SEM) images (1000×) and particle size distributions of the powders were shown in Figure 1.

### 2.2. Experimental Process Design and Related Parameters

The optimum SLM process parameters of individual materials have been studied in previous experiments, and the optimized results were shown in Table 4.

At present, connection strategies of dissimilar materials forming include direct joining, gradient path method, and intermediate section method. Direct joining refers to the method of directly forming another material on one material. The gradient path method refers to that although the transition part is added between the two materials, a third material is not introduced in the transition part. The connection is improved by changing process parameters and other factors. The intermediate section method is to add a third material between the two to improve the connection. Direct joining is usually used where the difference between the physical and chemical properties of the two materials is relatively low while the intermediate section method is opposite. The gradient path method is usually used when the situation is in between. Considering that the physical and chemical properties of NiTi and CuSn10 are quite different, direct joining is useless. The intermediate section method requires complex process exploration, so the gradient path method was selected here for forming. That is, the gradient path method was used to combine the two materials into a part. The strategy of forming CuSn10 alloy after NiTi alloy was adopted in this study. This is mainly because when CuSn10 is formed first, the heat of the laser beam will be rapidly dissipated by CuSn10 with good thermal conductivity when NiTi is formed so that enough heat cannot be gathered to form NiTi with high quality. At the same time, due to that NiTi is very sensitive to heat, the small part of NiTi formed first is easy to become separate from CuSn10 matrix due to thermal stress. The substrate was composed of NiTi alloy so the first layer could be easily connected to the substrate. Direct forming was carried out after remelting once in this experiment. An orthogonal scanning strategy was adopted to form all samples. That is, the laser scanning directions of adjacent layers were perpendicular to each other. The reason why this scanning method was adopted was that different heat flow directions would refine the grain so that the strength could increase [24]. In this study, small cuboids of dissimilar materials were formed, and the analysis of microstructure, element diffusion, microhardness, and phases was conducted on them. The process parameters of the first formed 20 layers of CuSn10 were taken as variables, the influence of which on the interfacial properties was studied. Considering that there was no relevant experiment on forming NiTi/CuSn10 dissimilar materials by SLM, the number of variables was set to only two. Laser power and scanning speed with small fluctuation were adopted to obtain different energy density, trying to preliminarily conclude how the forming of NiTi/CuSn10 dissimilar materials is affected by energy density. The calculation formula of energy density was shown in (1), in which *E* is energy density, *P* is laser power, *v* is scanning speed, *h* is scanning space, and *t* is layer thickness. The selected parameters were shown in Table 5. The scanning space is *h* = 0.08 mm and the layer thickness is *t* = 0.03 mm. Except for these layers, other parts were formed with the parameters in Table 4.

(1) $$E=\frac{P}{vht}$$

**Figure 2:**
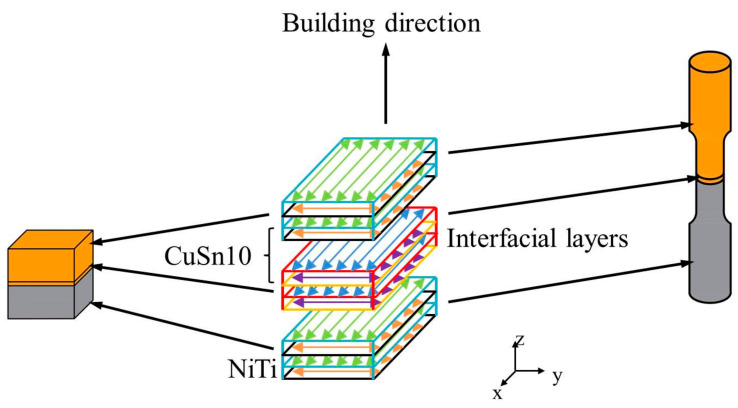
Diagram of forming the small cuboids and the tensile samples.

**Figure 3:**
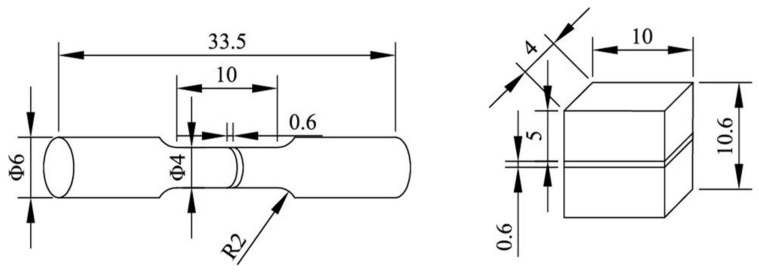
Sizes of the small cuboids and the tensile samples.

Small cuboids were 10 mm × 4 mm × 10 mm in size. A total of 5-mm high NiTi part was formed first, then the NiTi powders were cleared quickly, and CuSn10 powders were put in the powder chamber. Next 20 layers of CuSn10 (0.6 mm) was formed on the formed NiTi playing the role of transient inter-alloys. Finally, a 4.4-mm high CuSn10 was formed on the formed CuSn10. Tensile samples for the bonding strength test were formed in the same way as these small cuboids. For comparison, NiTi and CuSn10 single alloy tensile samples of the same size were formed with the parameters shown in Table 4. Figure 2 shows the diagram of forming the small cuboids and the tensile samples. Each parameter was used to fabricate three tensile samples to avoid occasionality. Figure 3 shows the sizes of the small cuboids and the tensile samples.

### 2.3. Microscopic Feature

The cuboids were removed from the substrate using Wire Electrical Discharge Machining. The sides of the cuboids were sanded and polished. After polishing, the metallurgical defects at the interface of the dissimilar materials were observed by optical microscope (OM) (DMI3000M, Leica, Weztlar, Germany). After observation, samples were etched for 2 s with a mixed solution (6 g FeCl_3_ + 10 mL HCl + 150 mL H_2_O) and 10 s with another mixed solution (1 mL HF + 2 mL HNO_3_ + 10 mL H_2_O) to further observe the microscopic features. The microstructure at the interface was observed with SEM (QUANTA250, FEI, Hillsboro, OR, USA). Energy Dispersive Spectroscopy (EDS) (Noran System 7, Thermo Fisher Scientific, Waltham, MA, USA) was used for point scanning along the direction perpendicular to the interface to analyze the element distribution at the interfacial region. X-ray diffraction (XRD) tests were conducted in a step-by-step manner to analyse phases at the interface: step scan 2θ = 20–90°, scanning speed = 0.6°/min, step width = 0.01°. The results were analyzed with jade 6.0.

### 2.4. Mechanical Properties

Vickers hardness tester (Wilson VH1202, Buehler, Lake Bluff, IL, USA) was used to measure the microhardness from CuSn10 alloy to NiTi alloy at the interfaces of the cuboids at 50 μm intervals. Tensile tests were performed on a universal testing machine (CMT5105, SUST) at a speed of 0.1 mm per min to measure the interfacial bonding strengths and the tensile strength of CuSn10 and NiTi single alloy.

## 3. Results and Discussion

### 3.1. Microstructure of NiTi/CuSn10

**Figure 4:**
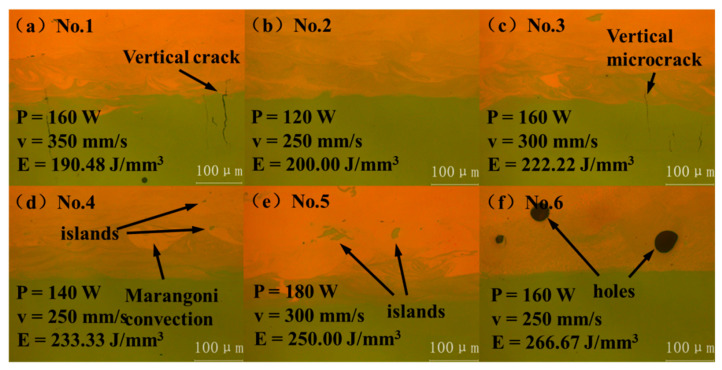
Optical micrographs of interfaces: Sample (**a**) No.1; (**b**) No.2; (**c**) No.3; (**d**) No.4; (**e**) No.5; (**f**) No.6.

The microstructure of these cuboids observed with the OM was shown in Figure 4. When *E* = 190.48 J/mm^3^, due to the fast-scanning speed, the cooling speed was also fast. So, a large temperature gradient was formed along the scanning route, thus a large thermal stress was formed. Cracks caused by thermal stress did not originate in CuSn10 alloy due to its good ductility. On the contrary, vertical cracks originated in the process of thermal stress release in NiTi alloy the ductility of which is relatively worse and extended to CuSn10 alloy, as shown in Figure 4a [25,26]. When *E* = 200.00 J/mm^3^, the scanning speed and energy density were moderate, so the interface quality was good and no obvious defects were observed, as shown in Figure 4b. When *E* = 222.22 J/mm^3^, vertical microcracks also occurred due to high scanning speed, as shown in Figure 4c. When *E* = 233.33 J/mm^3^ and 250.00 J/mm^3^, it can be seen that with the increase of energy density, movement of the molten pool became more intense and Marangoni convection appeared. The two materials penetrated each other, and the generation of an island can be observed, as shown in Figure 4d,e [7]. When *E* = 266.67 J/mm^3^, movement of the molten pool was further intensified and the gas in the building chamber was involved in the part, forming large pores, as shown in Figure 4f. Two types of defects, pores, and vertical cracks appeared in the process of gradually increasing energy density. While the cracks in the direction perpendicular to the interface generally have little effect on the bonding strength of the joint, samples No.1–No.5 were selected for the follow-up tests [5].

**Figure 5:**
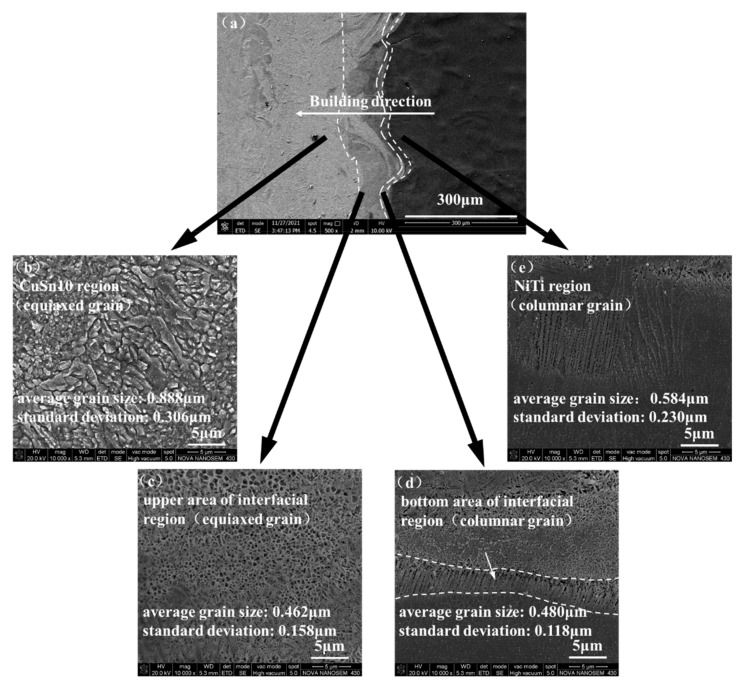
The microstructure of (**a**) NiTi/CuSn10 interface (500×); (**b**) CuSn10 region (10,000×); (**c**) upper area of interfacial region (10,000×); (**d**) bottom area of interfacial region (10,000×); (**e**) NiTi region (10,000×).

The microstructure of samples No.1–No.5 was observed with SEM after etching. Sample No. 1 was selected as a typical sample for microstructure analysis. In Figure 5a, the NiTi/CuSn10 interface was divided into four zones: CuSn10 region, upper area of interfacial region, bottom area of the interfacial region, and NiTi region, as shown in Figure 5b–e. CuSn10 region was composed of equiaxed grains. Upper area of the interfacial region was also composed of equiaxed grains, and both NiTi and CuSn10 could be seen in this zone. Bottom area of the interfacial region was composed of columnar grains, which formed along the molten pool boundary. Columnar grains formed due to the orientation of the thermal gradient. They were formed from CuSn10 to NiTi (along the direction of heat dissipation), playing the role of a stiffener. That is, the columnar grains were embedded into NiTi alloy, making the bonding firmer. So, it can be concluded that the interfacial bonding properties may be improved. NiTi region was also composed of columnar grains. The average grain size and standard deviation for each microstructure zone were listed in Figure 5. The width of columnar grain was regarded as its grain size. It can be seen that the average grain sizes of upper area of the interfacial region and bottom area of the interfacial region were smaller than those of CuSn10 region and NiTi region. A possible explanation is that the mixing of multiple elements in the interfacial region may promote a large increase in the number of crystal nuclei and significantly refine the grains [5]. This may also improve the interfacial bonding properties.

### 3.2. The Element Diffusion at the NiTi/CuSn10 Interface

**Figure 6:**
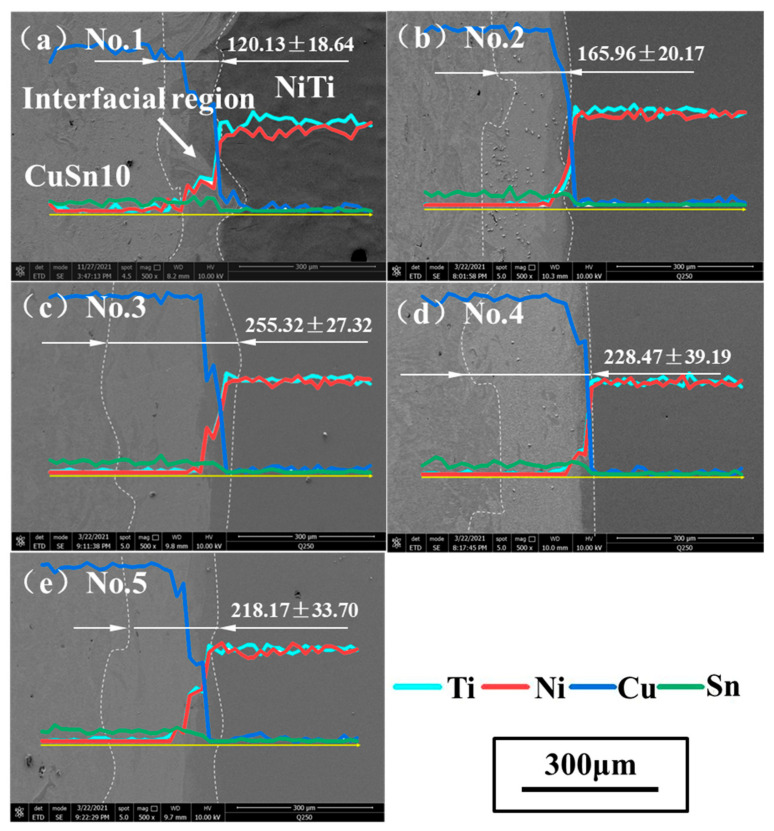
Interfacial SEM images of sample (**a**) No.1; (**b**) No.2; (**c**) No.3; (**d**) No.4; (**e**) No.5.

The samples observed with SEM were shown in Figure 6 (500×). The widths of interfacial regions of five samples were measured and the results were indicated in Figure 6. The maximum was 255.32 ± 27.32 μm while the minimum was 120.13 ± 18.64 μm.

It can be seen that the width of the interfacial region tended to increase with the increase of energy density. This was mainly because as the energy density increased, the molten pool movement became more violent and thus promoting the mixing of these two alloys. However, this promoting effect had a certain range. When the energy density was higher than a certain value, it can be seen that interfacial region thickness did not increase and tended to be stable. When it comes to a single parameter, it can be seen when comparing samples No.1 and No.3 that when the laser power was constant, the higher the scanning speed, the lower interfacial region thickness was. This was because the diffusion time was shorter when the scanning speed was higher [5]. It can also be seen when comparing samples No.2 and No.4 that when the scanning speed was constant, the larger the laser power and the higher the interfacial region thickness was. This was because the larger the laser power was, the larger the energy input was; thus the molten pool was deeper. Furthermore, the element distribution at the interface was analyzed. The main elements Ni, Ti, Cu, and Sn of the two alloys were taken as the analysis objects. The chemical composition was measured at a point every 2.88 μm along the building direction and 50 points were selected for each sample. The distribution results of interface elements were obtained as shown in Figure 6. Due to the small content of Sn, the change trend was not obvious, but it can still be seen that the content decreased when crossing the interface. The content of Ni and Ti decreased continuously while the content of Cu increased continuously when crossing the interface, indicating that the element mixing at the interfacial region was relatively sufficient.

### 3.3. The Microhardness at the NiTi/CuSn10 Interface

**Figure 7:**
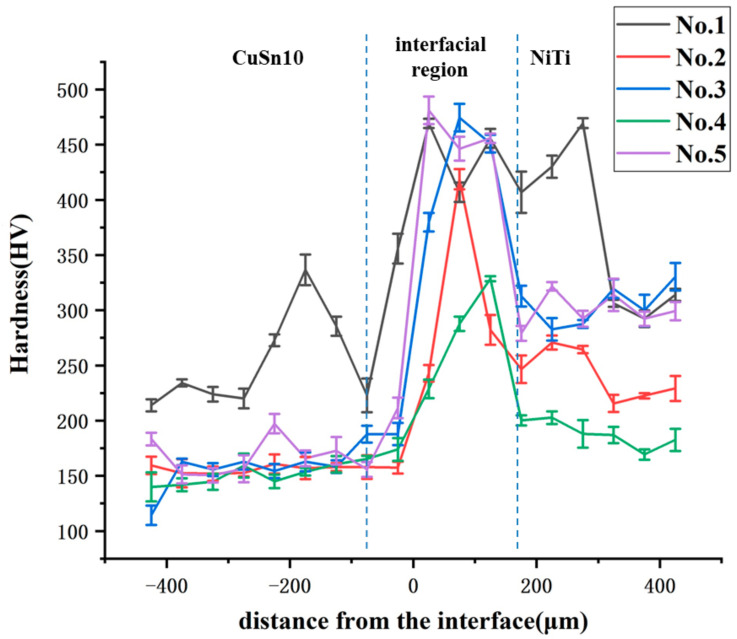
Microhardness change at the interface.

**Figure 8:**
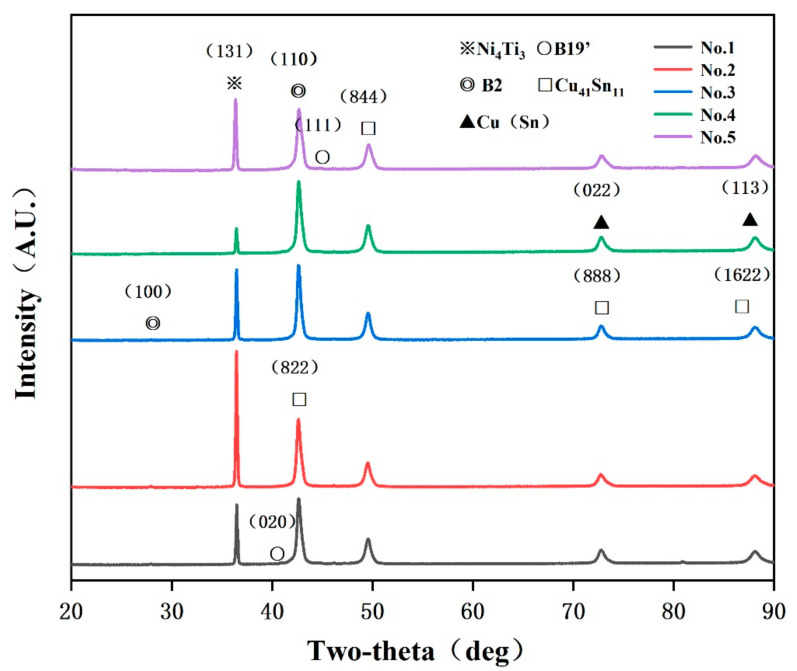
XRD pattern of the NiTi/CuSn10 interfaces.

The microhardness of each sample from CuSn10 alloy to NiTi alloy was measured at 50 μm intervals, and the results were shown in Figure 7. It can be seen that the microhardness was almost constant at first. Then it suddenly became larger at the interface, next it suddenly became smaller, and finally it became constant again. According to the change trend of microhardness, the area near the interface can be divided into CuSn10 zone, interfacial region, and NiTi zone. It is worth noting that the microhardness of the interfacial region was much higher than that of both sides. Sing et al. [27] found intermetallic compounds at the interface, which can make microhardness become higher. Zhou et al. [22] found that less brittle and hard Cu and Ni rich intermetallics (i.e., Cu3Ti, Ni3Ti) and more brittle Ti-rich intermetallics (i.e., Ti2Cu, Ti2Ni) could be formed in the process of dissimilar laser welding of NiTi and copper. This indicated that intermetallic compounds were possible to form between Cu alloy and NiTi alloy. Therefore, it can be considered that some kind of intermetallic compound was formed at the interface. In order to test the existence of interfacial intermetallic compound, XRD analysis was conducted on the interfaces of the five samples, and the experimental results were shown in Figure 8.

First, the related phases of NiTi alloy and CuSn10 alloy were determined by phase identifying. Cu(Sn) and Cu41Sn11 were phases that often appeared in SLM CuSn10 alloy while B2 and B19′ were two phases of NiTi alloy. After this, it was found that a new phase appeared, which was found to be Ni_4_Ti_3_ after searching and comparing. This was because the scanning speed and laser power used at the interface were not high so the heating and cooling rate of the molten pool was low, which was conducive to the formation of Ni_4_Ti_3_ [28]. Ni_4_Ti_3_ is a very fine phase, which can produce dispersion strengthening and increase the strength of the sample interface [29]. Therefore, the formation of Ni_4_Ti_3_ intermetallic compound played a key role in the increase of microhardness at the interface. At the same time, it can be inferred that the interfacial bonding performance had been improved.

### 3.4. Bonding Strength Test of NiTi/CuSn10 Interface

**Figure 9:**
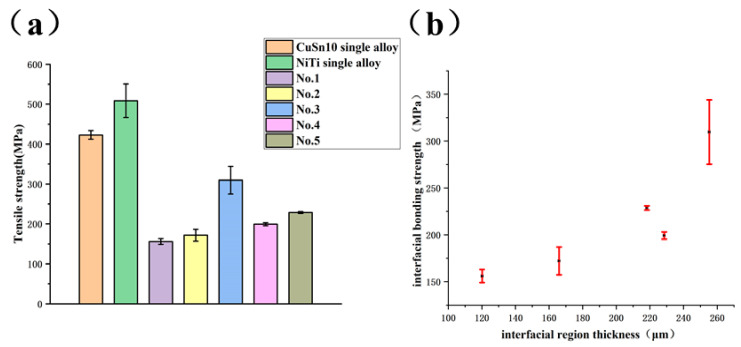
(**a**) The interfacial bonding strengths of different samples compared with the tensile strength of CuSn10 and NiTi single alloy (**b**) relationship between interfacial bonding strength and interfacial region thickness.

The interfacial bonding strength between NiTi and CuSn10 has a great influence on the properties of the whole part. In order to study the bonding strength of NiTi/CuSn10, tensile tests of dissimilar materials samples were conducted, and the results were shown in Figure 9a.

The strengths of NiTi and CuSn10 single alloy formed vertically by SLM had been optimized and were used as the control groups with values of 508.6 ± 42.2 MPa and 422.9 ± 10.8 MPa, respectively. All the tensile parts broke at the interface of the two materials. It can be seen that sample No.1 had the worst interfacial bonding strength (156.0 ± 7.0 MPa), equivalent to 37% of that of CuSn10 single alloy. Sample No.3 had the best interfacial bonding strength (309.7 ± 34.4 MPa), equivalent to 75% of that of CuSn10 single alloy. Based on previous results, although there were vertical microcracks at the interface of process parameters corresponding to sample No.3, the interfacial bonding properties of sample No.3 was still better than that of other samples with no observed defects. This indicated that the vertical microcracks did not have a significant impact on interfacial bonding properties. The relationship between interfacial bonding strength and interfacial region thickness was shown in Figure 9b. It can be obtained from Figure 9b that the interfacial bonding strength was positively correlated with interfacial region thickness. This was because the higher the interfacial region thickness was, the more solid metallurgical bonding could be formed at the interface so that the interfacial bonding strength would be higher. Columnar grains at the interface and grain refinement in the interfacial region played a strengthening role. Based on the phase analysis, the improvement of interfacial bonding strength was also related to the formation of fine Ni_4_Ti_3_ strengthening phase.

**Figure 10:**
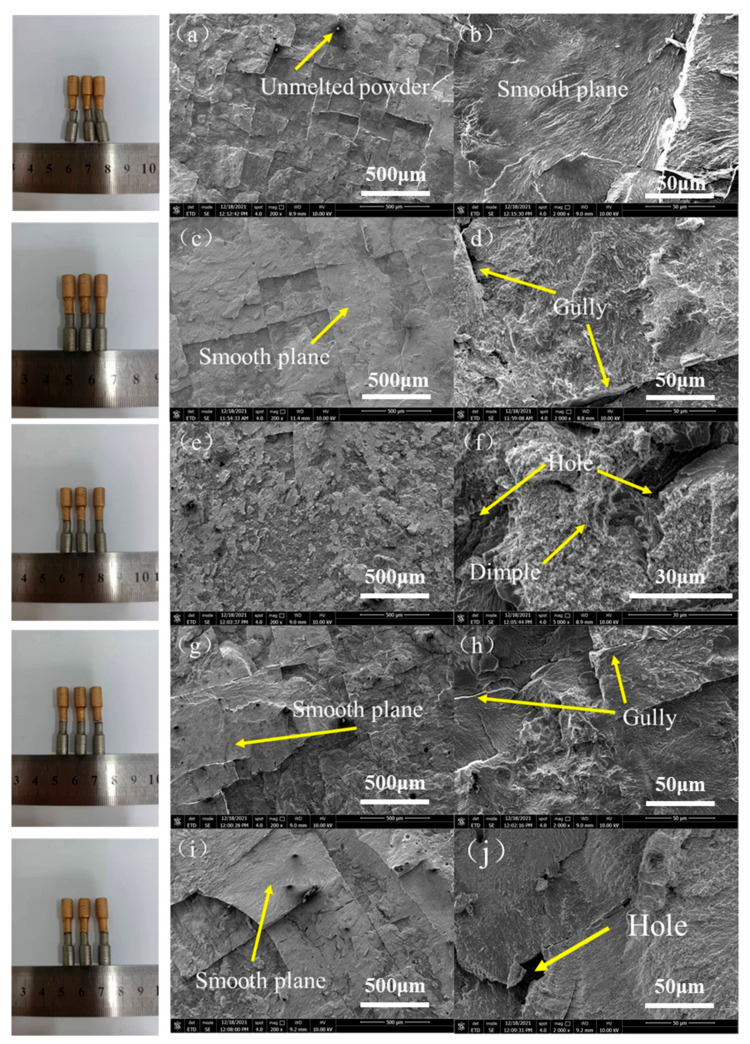
SEM images of fracture of sample (**a**) No.1 (200×); (**b**) No.1 (2000×); (**c**) No.2 (200×); (**d**) No.2 (2000×); (**e**) No.3 (200×); (**f**) No.3 (5000×); (**g**) No.4 (200×); (**h**) No.4 (2000×); (**i**) No.5 (200×); (**j**) No.5 (2000×).

Low magnification and high magnification SEM images were taken for each fracture, as shown in Figure 10.

At low magnification (200×), it can be seen that the fracture was very flat, and some unmelted metal powder can be seen occasionally, which indicated a brittle fracture as shown in Figure 10a. The whole fracture was divided into many small planes. At high magnification (2000×), some holes due to the gas not escaping and gullies between planes due to the existence of vertical cracks in these samples can be seen. On account of brittle fracture, these samples cannot achieve strength comparable to that of a single metal material. However, it is worth noting that a few dimples can be found in the fracture of sample No.3 at high magnification (5000×); that is, the sample had certain ductility. This indicated that a sample could have good interfacial bonding properties under the strengthening of columnar grain at the interface, grain refinement in the interfacial region and Ni_4_Ti_3_ with appropriate content.

## 4. Conclusions

The combination of different materials by SLM makes it possible to combine the excellent properties of different materials. In this paper, the selective laser melting technology was used to form CuSn10 alloy on the formed NiTi alloy. The microstructure, element diffusion, microhardness change, and phases at the interface were studied. Tensile parts were formed to test the interfacial bonding properties, and the conclusions were drawn as follows:

(1) Different interfacial structures can be obtained by adjusting the forming energy density input of the interface by adjusting process parameters. Columnar grains can form along the boundary of molten pool in the interfacial region and can effectively improve the interfacial bonding. Grain refinement in the interfacial region can also play a strengthening role.
(2) In a certain range, the width of the interfacial region increases with the increase of energy density. Beyond a certain range, the width of the interfacial region remains basically unchanged. The wider the interfacial region is, the stronger metallurgical bonding can be formed at the interface.
(3) The formation of N_4_Ti_3_ strengthening phase improves the microhardness of the interface, making the microhardness of the interface significantly higher than that of both sides. The interfacial bonding strength is different under different process parameters. Under the optimized process parameters, the maximum strength can reach 309.7 ± 34.4 MPa.

In general, the interfacial bonding is still weak, but on the premise of the large gap between the physical and chemical properties of CuSn10 and NiTi alloy, it can be said that the interfacial bonding strength has reached a good level with a value of 309.7 ± 34.4 MPa. This direction still has good application prospects. In addition, how different process parameters affect the interfacial Ni_4_Ti_3_ strengthening phase, columnar grain, and grain refinement, thus affecting the interfacial bonding strength still needs further quantitative research.

## Figures and Tables

**Table 1:** Main equipment parameters of SLM Dimetal-100H device.

Item	Parameter
Building size/mm	100 × 100 × 100
Laser	500 W infrared ytterbium doped fiber laser
Wavelength/nm	1075
Beam Quality Factor	M^2^ ≤ 1.1
Focus beam size/μm	40–60
Layer thickness/μm	20–100
Processing speed/(cm^3^/h)	4–20

**Table 2:** Chemical composition of equiatomic NiTi50 powder.

Element	Ni	Fe	C	O	N	Ti
Proportion (wt.%)	54.5	0.0074	0.006	0.0607	0.0018	Bal.

**Table 3:** Chemical composition of CuSn10 powder.

Element	Cu	Sn	Others
Proportion (wt.%)	Bal.	10.11	<0.06

**Table 4:** Optimum SLM process parameters of NiTi alloy and CuSn10 alloy.

Item	NiTi	CuSn10
Laser power (W)	140	160
Scanning speed (mm/s)	500	300
Scanning space (mm)	0.08	0.08
Layer thickness (mm)	0.03	0.03
Relative density (%)	98.83	99.95

**Table 5:** Process parameters of interlayers.

Experiment No.	Laser Power/(W)	Scanning Speed/(mm/s)	Energy Density/(J/mm^3^)
1	160	350	190.48
2	120	250	200.00
3	160	300	222.22
4	140	250	233.33
5	180	300	250.00
6	160	250	266.67
